# The AS-PAD Score Predicts Significant Peripheral Artery Disease and Is Associated with Femoral Access-Site Closure Device Failure in Patients Undergoing TAVI

**DOI:** 10.3390/medicina62071293

**Published:** 2026-07-04

**Authors:** Uttban Gohman, Mohamad Amer Nashtar, Paula Liedtke, Obayda Azizy, Jörn Trippe, Justus Strauch, Rolf Alexander Jánosi, Panagiotis Iliakis, Kyriakos Dimitriadis, Konstantinos Tsioufis, Ali Canbay, Ingo Schmitz, Martin Steinmetz, Polykarpos Christos Patsalis

**Affiliations:** 1 Division of Cardiology, Angiology and Internal Emergency Medicine, Department of Medicine, Knappschaft Kliniken University Hospital Bochum, Ruhr University Bochum, 44892 Bochum, Germany; uttban.gohman@knappschaft-kliniken.de (U.G.); mohamad.nashtar@knappschaft-kliniken.de (M.A.N.); paula.liedtke@rub.de (P.L.); dr.azizy@outlook.com (O.A.); joern.trippe@knappschaft-kliniken.de (J.T.); panayiotisiliakis@gmail.com (P.I.); 2 Institute of Molecular Immunology, Ruhr University Bochum, 44801 Bochum, Germany; ingo.schmitz@rub.de; 3 Department of Cardiac and Thoracic Surgery, University Hospital Bergmannsheil, Ruhr University Bochum, 44789 Bochum, Germany; justus.strauch@bergmannsheil.de; 4 Department of Cardiology, Angiology and Electrophysiology, Heart Center Duisburg, 47169 Duisburg, Germany; alexander.janosi@evkln.de; 5 First Department of Cardiology, School of Medicine, National and Kapodistrian University of Athens, Hippokration General Hospital, 11527 Athens, Greece; dimitriadiskyr@yahoo.gr (K.D.); ktsioufis@gmail.com (K.T.); 6 Division of Hepatology and Gastroenterology, Department of Medicine, Knappschaft Kliniken University Hospital Bochum, Ruhr University Bochum, 44892 Bochum, Germany; ali.canbay@rub.de

**Keywords:** aortic stenosis, peripheral artery disease, transcatheter aortic valve implantation, vascular complications, Lipoprotein(a), diabetes mellitus

## Abstract

*Background and Objectives*: Patients with severe aortic stenosis (AS) frequently present with concomitant peripheral artery disease (PAD), which complicates transcatheter aortic valve implantation (TAVI) and increases the risk of vascular complications. Early identification of significant PAD may facilitate optimized procedural planning and risk stratification. This study aimed to develop and internally validate a clinical score for estimating the likelihood of significant PAD in patients with severe AS undergoing TAVI evaluation and to exploratorily assess its association with femoral access-site closure-device failure after TAVI. *Materials and Methods*: In this retrospective study, 138 patients with severe AS (124 undergoing TAVI) were evaluated. Logistic regression analyses identified independent predictors of significant PAD (≥50% stenosis), which were incorporated into the AS-PAD Score. The Score integrates age >75 years, diabetes mellitus, BMI ≥25 kg/m^2^, Lp(a) >50 mg/dL (105 nmol/L), and Fontaine clinical stage. Model performance was assessed using receiver operating characteristic (ROC) analysis and internally validated using bootstrap resampling and stratified five-fold cross-validation. Vascular complications were adjudicated according to VARC-3 criteria. *Results*: The AS-PAD Score demonstrated good discriminatory performance, with an AUROC of 0.804. Advanced age (OR 2.43, 95% CI:1.05–5.70; *p* = 0.039) and diabetes (OR 3.18, 95% CI:1.29–7.83; *p* = 0.012) were independently associated with significant PAD, while BMI ≥25 kg/m^2^ showed an inverse association. Elevated Lp(a) (>50 mg/dL) remained independently associated with significant PAD. Internal validation using bootstrap resampling and stratified five-fold cross-validation demonstrated consistent model performance. Femoral access-site closure-device failure occurred more frequently among patients classified as intermediate or high risk by the AS-PAD Score compared with low-risk patients. *Conclusions*: The AS-PAD Score may serve as an adjunctive clinical tool for estimating the likelihood of significant PAD in patients with severe AS undergoing TAVI evaluation. Its association with femoral access-site closure-device failure should be interpreted as exploratory and hypothesis-generating. Further external validation is required to confirm its role alongside standard CT-based vascular assessment.

## 1. Introduction

Peripheral artery disease (PAD) is a common, progressive atherosclerotic condition affecting an estimated 200 million individuals worldwide [1]. Its prevalence increases with age and with cardiovascular risk factors such as diabetes mellitus, smoking, and chronic kidney disease [2].

In patients with severe aortic stenosis (AS) undergoing transcatheter aortic valve implantation (TAVI), the presence of PAD has been linked to increased mortality, higher rates of procedural complications [3,4,5], and limited vascular access options [6,7]. Early identification of PAD is therefore essential, as it significantly influences periprocedural planning, individualized treatment strategies, and postprocedural outcomes [8]. However, screening for PAD in patients with AS remains suboptimal [9].

The conceptual framework and methodological design of the Aortic Stenosis–Peripheral Artery Disease (AS-PAD) Score were inspired by the recently developed Aortic Stenosis–Coronary Artery Disease (AS-CAD) Score, which stratifies the likelihood of significant coronary artery disease in patients with severe AS undergoing TAVI [10]. Analogous to the AS-CAD approach, we developed the AS-PAD Score based on routinely available clinical parameters, aiming to support clinical decision-making by enabling early PAD risk stratification. This study presents the derivation, structure, and internal validation of the AS-PAD Score in a cohort of patients with severe aortic stenosis evaluated for either TAVI or surgical aortic valve replacement (sAVR).

As TAVI expands into younger and lower-risk populations, the ability to identify patients with significant PAD and potential femoral access-site management challenges becomes increasingly important [11,12]. Reliable preprocedural risk assessment may support individualized care strategies and standardized procedural workflows in contemporary TAVI practice.

## 2. Methods

### 2.1. Study Design and Patient Enrollment

This single-centre cohort study included 138 patients with severe AS who were retrospectively evaluated between 2020 and 2024 for TAVI (*n* = 124) or sAVR. The study was approved by the local Ethics Committee of Ruhr University Bochum/Knappschaft Kliniken University Hospital Bochum, Germany (approval code: 20-6962-§ 23b; approval date: 17 March 2023). Severe AS was defined according to the 2021 ESC/EACTS Guidelines for the management of valvular heart disease, i.e., aortic valve area (AVA) ≤1.0 cm^2^, a mean transvalvular gradient ≥40 mmHg, or a peak velocity ≥4.0 m/s [13].

Patients were excluded if they had a bicuspid aortic valve, active malignancy with limited life expectancy (<12 months), acute vascular syndromes (e.g., myocardial infarction, stroke, or limb ischemia), or contraindications to TAVI or sAVR. The study was conducted in accordance with the Declaration of Helsinki and approved by the institutional ethics committee.

PAD was defined, according to the 2017 ESC/ESVS guideline on peripheral arterial diseases and the 2024 ESC guideline on management of peripheral arterial and aortic diseases as the presence of atherosclerotic stenosis in at least one peripheral artery (e.g., iliac, femoral, popliteal, or tibial) detected via duplex ultrasonography or contrast-enhanced computed tomography angiography (CTA) or magnetic resonance angiography (MRA) [14,15]. Significant PAD was defined as luminal narrowing of ≥50%, consistent with the 2016 AHA/ACC Guidelines for the management of lower extremity peripheral artery disease [16]. Clinical symptoms were graded using the Fontaine classification (stage I–IV) based on walking distance, pain at rest, and presence of tissue loss.

### 2.2. TAVI and Assessment of Vascular Complications

TAVI was performed via the transfemoral approach in all patients. Procedural success, including the evaluation of vascular complications, was assessed according to the Valve Academic Research Consortium-3 (VARC-3) criteria [17].

Management of suboptimal vascular closure and/or closure device failure was performed using one of the following interventional techniques: placement of an additional ProGlide device (Abbott Vascular, Santa Clara, CA, USA), additional use of an Angio-Seal 8F closure device (Terumo Medical Corporation, Somerset, NJ, USA), implantation of a MANTA 18F vascular closure device (Teleflex, Wayne, PA, USA), or deployment of a covered stent using a crossover technique with Fluency self-expanding grafts (BD, Franklin Lakes, NJ, USA). No access-site complication required surgical intervention. For the exploratory post-TAVI analysis, femoral access-site closure-device failure was defined as partial or complete failure of percutaneous access-site closure requiring additional percutaneous or endovascular management. Events were considered VARC-3 minor vascular complications only when closure-device failure required alternative or additional treatment without death, VARC type ≥ 2 bleeding, limb or visceral ischemia, irreversible neurological impairment, or need for surgical vascular repair. This endpoint was distinguished from major PAD-related vascular complications, including limb ischemia, vessel perforation, major dissection, irreversible neurological impairment, or surgical vascular repair.

### 2.3. Patient Characteristics

Demographic data, cardiovascular risk factors, medical history, and laboratory values were collected. Risk factor variables included: sex, age, BMI (kg/m^2^), arterial hypertension, diabetes mellitus, chronic kidney disease, current or former smoking, hypercholesterolemia, and hyperlipidaemia. Laboratory values included high-sensitivity C-reactive protein (hs-CRP), interleukin-6 (IL-6), and lipoprotein(a) [Lp(a)].

### 2.4. Development and Mathematical Validation of AS-PAD Score

The AS-PAD Score was developed through a multi-phase process involving univariable logistic regression for initial screening, followed by multivariable logistic regression and internal validation. In the first phase, univariable logistic regression was applied to all cardiovascular risk variables to identify potential candidates associated with significant PAD (≥50% stenosis). Variables with a *p* < 0.10 were entered into a multivariable logistic regression model, in which a backward stepwise elimination method based on the Wald statistic was used to identify the final set of independent predictors. For score construction, Lp(a) was dichotomized, with elevated Lp(a) defined as >50 mg/dL (105 nmol/L), in line with contemporary ESC/EAS recommendations indicating increased cardiovascular risk above this threshold [18]. Fontaine stage was included as a clinical measure of symptom severity.

The variables were integrated into a point-based score according to their effect size (odds ratio). The final AS-PAD Score included age >75 years, diabetes mellitus, BMI ≥ 25 kg/m^2^ (inverse association), Lp(a) > 50 mg/dL and Fontaine stage. The total score ranges from −1 to 10 and was calculated individually for each patient.

To assess model performance, the total score was used as a continuous predictor. Model discrimination was evaluated using receiver operating characteristic (ROC) analysis, and an optimal cut-off was defined using the Youden index. Internal validity was assessed using two complementary approaches, both conducted on the same dataset. First, bootstrap-based internal validation with optimism correction was performed using 1000 bootstrap iterations. In each bootstrap sample, a logistic regression model using the total AS-PAD Score as a continuous predictor was refitted. Model discrimination was assessed by comparing the apparent area under the curve (AUC) with the optimism-corrected AUC. Calibration was assessed using the Brier score, calibration intercept, calibration slope, and a 95% calibration belt. Because calibration was assessed in the derivation cohort, these analyses were interpreted as apparent calibration. Second, stratified five-fold cross-validation was performed. Patients were assigned to five approximately equal folds while preserving the proportion of significant PAD events across folds. In each iteration, a logistic regression model using the total AS-PAD Score as a continuous predictor was fitted in four folds and evaluated in the held-out fold. Fold-specific AUCs were reported, and the mean AUC with standard deviation and median AUC with interquartile range were calculated. In addition, a pooled cross-validated AUC was calculated from all out-of-fold predicted probabilities, with the 95% confidence interval estimated using bootstrap resampling.

Clinical utility was evaluated using decision-curve analysis. Net benefit was calculated across threshold probabilities by comparing the AS-PAD Score model with two reference strategies: treating all patients as having significant PAD and treating no patients as having significant PAD. Predicted probabilities were derived from logistic regression using the total AS-PAD Score as a continuous predictor. Because decision-curve analysis was performed in the derivation cohort, it was interpreted as an assessment of apparent clinical utility.

### 2.5. Statistical Analysis

Statistical significance was defined as a two-tailed *p*-value < 0.05. Continuous variables are presented as mean ± standard deviation or median with interquartile range, depending on data distribution. Categorical variables are summarized as absolute counts and percentages. Missing values were not imputed, and analyses were performed using the available observations for the respective variable. All analyses were performed using IBM SPSS Statistics (version 29.0.2.0 (20); IBM Corp., Armonk, NY, USA) and R software (version 4.6.0; R Foundation for Statistical Computing, Vienna, Austria). Graphical representations were created using GraphPad Prism (version 10.5; GraphPad Software, San Diego, CA, USA) and R software.

## 3. Results

### 3.1. Patient Cohort Analysis

A total of 138 patients with echocardiographically confirmed severe AS were included in the analysis. The mean age was 81.2 years (±8.01), and 76 patients (55.07%) were female. Arterial hypertension was present in 89.86% of patients, diabetes mellitus in 36.96%, and chronic kidney disease in 23.19%. A total of 59.42% had a body mass index (BMI) ≥25 kg/m^2^, and 57.25% were active or former smokers. PAD of any grade was present in 88 patients (63.77%), while 35 patients (25.36%) had a significant PAD, defined as ≥50% stenosis in at least one lower extremity artery. In 92.5% of patients with PAD, multisegmental involvement was present, irrespective of score stratification. Among patients with clinically significant PAD, 97% demonstrated multisegmental disease. Baseline characteristics are summarized in Table 1.

### 3.2. Identification of PAD Predictors

To identify predictors of significant PAD in this high-risk population, uni- and multivariable logistic regression analyses were performed. In univariable logistic regression, age > 75 years was significantly associated with the presence of significant PAD (OR = 2.51, *p* = 0.031), as were diabetes mellitus (OR = 3.01, *p* = 0.005) and elevated Lp(a) levels (OR = 1.68, *p* = 0.028). In the final multivariable model derived by backward stepwise elimination, age >75 years remained an independent predictor (OR = 2.43, *p* = 0.039), alongside diabetes mellitus (OR = 3.18, *p* = 0.012) and elevated Lp(a) (OR = 1.42, *p* = 0.044). Among the tested age thresholds (>65, >70, >75 years), only age > 75 demonstrated a statistically significant association with significant PAD in both univariable and multivariable models. This threshold also corresponded to the optimal ROC cut-off based on the Youden index and was therefore selected for inclusion in the AS-PAD Score, as it balanced statistical robustness with clinical usability. A BMI ≥ 25 kg/m^2^ was inversely associated with significant PAD (OR = 0.34, *p* = 0.016). Lastly, the Fontaine stage was incorporated directly into the score structure to reflect PAD severity. The selected predictors and their respective statistical associations are summarized in Table 2.

### 3.3. Composition of Clinical Score

Based on these findings, a five-component clinical score was constructed to estimate the likelihood of significant PAD. Variables were weighted according to their effect size and clinical interpretability. To enhance bedside applicability, the dichotomized variable age >75 years was chosen and assigned with 2 points. Diabetes mellitus received 3 points. A BMI ≥25 kg/m^2^, was assigned with −1 point. Lp(a) >50 mg/dL (105 nmol/L) was included with 1 point, and clinical PAD severity as classified by Fontaine stage contributed 0 to 4 points. The resulting AS-PAD Score thus ranged from −1 to 10 and was calculated for each patient.

**Figure 1:**
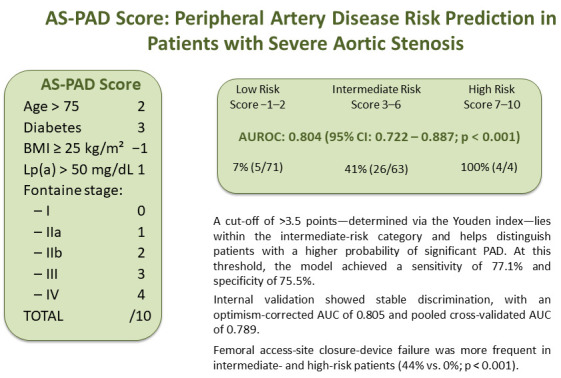
Visual representation of the five components of the AS-PAD Score and corresponding point allocation. The score ranges from −1 to 10 points and enables classification into three risk groups based on total score.

To evaluate its discriminatory capacity, patients were stratified into three risk groups: low (−1–2 points), intermediate (3–6 points), and high risk (7–10 points). The prevalence of significant PAD increased markedly across categories: 7% (5/71) in the low-risk group, 41.3% (26/63) in the intermediate group, and 100% (4/4) in the high-risk group. These findings are visually depicted in Figure 1.

**Figure 2:**
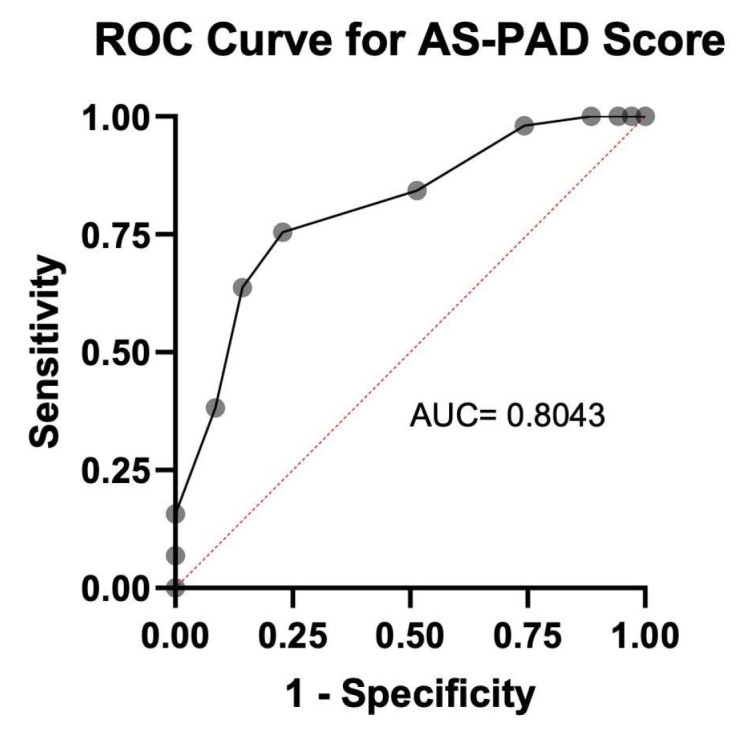
Receiver operating characteristic (ROC) curve illustrating the predictive ability of the AS-PAD risk score.The diagonal dashed line represents the reference line of a non-discriminatory classifier (AUC = 0.50). AUC = area under the curve.

Receiver operating characteristic (ROC) analysis confirmed strong discriminatory performance, with an area under the curve (AUC) of 0.804 (95% CI: 0.722–0.887). An optimal cut-off of >3.5 points was determined using the Youden index (0.526). This threshold lies within the intermediate-risk category and helped discriminate between lower- and higher-risk individuals within this group, as patients with a score of 3 showed a lower PAD prevalence (3/15; 20%) compared to those with scores > 3 (23/48; 48%). At this threshold, the model achieved a sensitivity of 77.1% and specificity of 75.5%. The ROC curve is shown in Figure 2.

**Figure 3:**
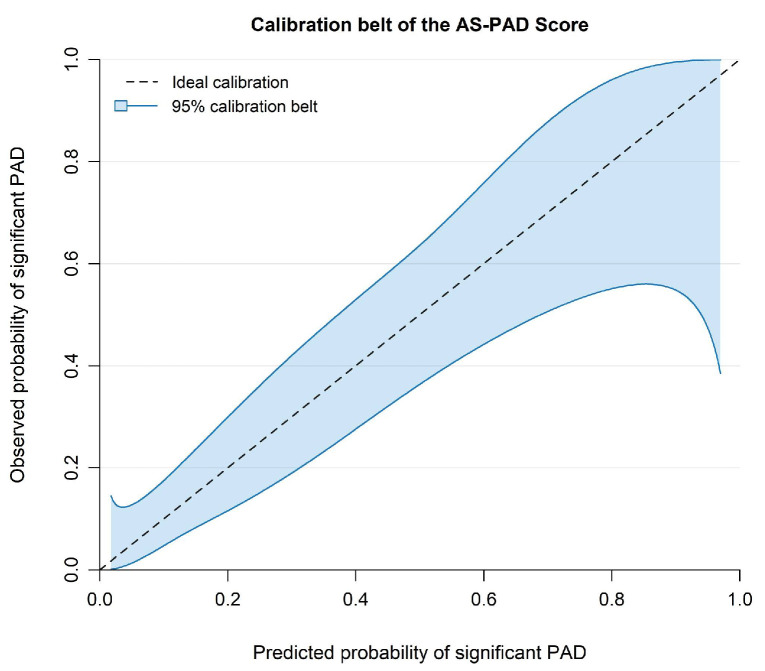
Calibration belt of the AS-PAD Score for significant peripheral artery disease. Calibration belt for the prediction of significant peripheral artery disease (PAD) using the AS-PAD Score in the derivation cohort. Predicted probabilities were derived from logistic regression using the total AS-PAD Score as a continuous predictor. The dashed diagonal line indicates perfect calibration, and the shaded area with boundary lines represents the 95% calibration belt.

Internal validation was conducted using two complementary methods. First, bootstrap-based internal validation with optimism correction was performed using 1000 bootstrap iterations. The apparent AUC of the AS-PAD Score in the full cohort was 0.804, and the optimism-corrected AUC was comparable at 0.805, suggesting no relevant optimism in discriminatory performance. The Brier score was 0.141, and the optimism-corrected Brier score was 0.146. Apparent calibration intercept and slope were 0.000 and 1.000, respectively; bootstrap-corrected calibration intercept and slope were 0.009 and 1.003, respectively (Table 3). Apparent calibration was additionally assessed using a 95% calibration belt, which did not indicate major deviation from ideal calibration. However, the width of the calibration belt reflects the limited number of events and should therefore be interpreted cautiously. The calibration belt is shown in Figure 3.

Second, stratified five-fold cross-validation was performed to assess out-of-sample discriminatory performance. Fold-specific AUC values ranged from 0.636 to 0.939. The mean cross-validated fold AUC was 0.794 ± 0.139, with a median AUC of 0.829 (IQR: 0.661–0.905). The pooled cross-validated AUC, calculated from all out-of-fold predicted probabilities, was 0.789 (95% bootstrap CI: 0.696–0.875). These findings indicate acceptable cross-validated discrimination, although the variability across folds reflects the limited number of events within each validation fold. The full results are shown in Table 4.

To assess the robustness of the AS-PAD Score, three separate sensitivity analyses were performed. These included exclusion of Fontaine stage, exclusion of BMI ≥ 25 kg/m^2^, and exclusion of patients who ultimately underwent surgical aortic valve replacement (sAVR) (*n* = 14). The resulting AUCs are presented in Table 5.

**Figure 4:**
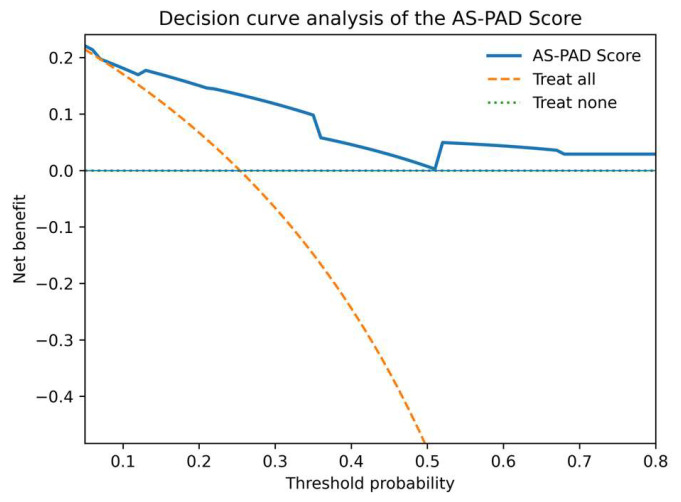
Decision-curve analysis of the AS-PAD Score. Decision-curve analysis evaluating the net benefit of the AS-PAD Score for predicting significant peripheral artery disease (PAD) across threshold probabilities. The AS-PAD Score was compared with treat-all and treat-none strategies. The score demonstrated a higher apparent net benefit across clinically plausible threshold probabilities, particularly between 5% and 50%.

Decision-curve analysis was performed to evaluate the apparent clinical utility of the AS-PAD Score. Across clinically plausible threshold probabilities, particularly between 5% and 50%, the AS-PAD Score showed a higher net benefit than both treat-all and treat-none strategies. For example, at threshold probabilities of 10%, 20%, 30%, 40%, and 50%, the net benefit of the AS-PAD Score was 0.181, 0.150, 0.118, 0.046, and 0.007, respectively. These findings suggest an apparent net benefit of the AS-PAD Score for risk stratification compared with non-selective strategies. However, because decision-curve analysis was performed in the derivation cohort, these results should be interpreted as apparent clinical utility and require confirmation in external cohorts. The decision curve is shown in Figure 4.

### 3.4. Exploratory Association Between the AS-PAD Score and Femoral Access-Site Closure-Device Failure Following TAVI

Among patients undergoing transfemoral TAVI, no major PAD-related access-site vascular complications, including limb ischemia, vessel perforation, major dissection, challenging catheter or sheath advancement requiring alternative access, or access-site complications requiring surgical vascular repair, were observed. The post-TAVI access-site endpoint in the present analysis therefore reflected minor femoral access-site management issues, primarily partial or complete failure of percutaneous access-site closure requiring additional interventional treatment.

Minor femoral access-site closure-device failure was more frequent in patients classified as intermediate or high risk according to the AS-PAD Score compared with low-risk patients (44% vs. 0%, *p* < 0.001). Twenty-two intermediate- or high-risk patients required additional interventional treatment for suboptimal closure or closure-device failure. Given the exploratory nature of this analysis and the limited number of events, these findings should be interpreted as an association between higher AS-PAD Score categories and femoral access-site closure-device failure rather than as evidence that the score independently predicts major PAD-related vascular complications.

## 4. Discussion

The present study introduces and internally validates the AS-PAD Score, a novel clinical tool designed to estimate the probability of significant peripheral artery disease (PAD) in patients with severe aortic stenosis (AS). Based on easily obtainable clinical parameters, the score demonstrated good discriminatory performance and acceptable apparent calibration, with internal validation supporting its consistency within the derivation cohort. Variable selection balanced statistical association and clinical relevance to enhance practical applicability.

Key predictors of significant PAD included age >75 years, diabetes mellitus and elevated Lp(a). Both increasing age and diabetes are established vascular risk factors for atherosclerosis [2,14], with mechanistic links particularly well described for diabetes [19]. Age was dichotomized at >75.0 years to facilitate bedside usability. This threshold aligns with previously described epidemiological data indicating that the prevalence of severe aortic stenosis rises sharply after the age of 75 [13,20]. Likewise, the prevalence of peripheral arterial disease increases substantially with age, with a marked rise beginning from 70 years onwards [2,14]. These findings support the integration of age >75 as a clinically and statistically justified threshold within the AS-PAD Score. Diabetes mellitus was included based on its consistent predictive value in this cohort, its clinical relevance in PAD populations [14], and its established role in promoting systemic atherosclerosis [19]. Elevated lipoprotein(a) [Lp(a)] was the only laboratory parameter consistently associated with significant PAD and was included in the AS-PAD Score as a clinically meaningful biomarker, supported by its established pathophysiological role in atherogenesis and its independent association with vascular risk [21].

Lastly, Fontaine stage was incorporated to reflect symptomatic PAD severity. As a standardized, guideline-endorsed classification system, it allows for the integration of symptom burden into the risk model using readily available clinical information [14].

Interestingly, a BMI ≥ 25 kg/m^2^ was found to be inversely associated with the presence of significant PAD in a statistically significant manner. On the one hand, epidemiological studies have demonstrated that an elevated body mass index (BMI) is associated with an increased risk of peripheral arterial disease (PAD). A recent analysis based on data from the National Health and Nutrition Examination Survey (NHANES) identified a higher BMI—particularly ≥35 kg/m^2^—as a significant risk factor for the development of PAD [22]. In addition, a pooled analysis of individual-level data from ten prospective cohort studies—including Framingham, NHANES, and others—also confirmed a positive association between elevated BMI and the incidence of PAD [23]. On the other hand, this inverse finding might align with the so-called ‘obesity paradox,’ which describes more favorable clinical outcomes among overweight patients [24,25], a phenomenon for which several explanations have been proposed, including greater physiological reserve, improved nutritional status, and reduced frailty [26]. However, the obesity paradox primarily concerns prognosis rather than disease prevalence, and the present study was not designed to investigate these mechanisms in detail. In this context, a recent comprehensive review by Lempesis et al. highlights the complexity of the relationship between obesity and PAD [27]. While obesity is widely recognized as a risk factor for atherosclerosis, the association between elevated BMI and PAD remains inconsistent across studies—some epidemiological data support a positive correlation, whereas others point to the so-called ‘obesity paradox’. Taken together, this finding should be interpreted with caution. The observed inverse association should not be interpreted as evidence of a protective biological effect of elevated BMI and requires confirmation in independent external cohorts.

The AS-PAD Score showed increasing PAD prevalence across risk categories, from 7.0% in the low-risk group to 41.3% in the intermediate-risk group. Although all four patients classified as high risk had significant PAD, this subgroup was very small (*n* = 4). Therefore, the observed 100% prevalence should be interpreted with caution and regarded as a descriptive observation rather than evidence of robust classification performance. Given the very small number of patients in this subgroup, the reproducibility of this observation remains uncertain and warrants evaluation in larger external cohorts. Notably, the overall prevalence of significant PAD in our cohort (35/138; 25.4%) was consistent with previous reports in TAVI populations, where significant PAD has been observed in up to one third of patients [3]. This risk score may thus serve as a practical decision point in clinical evaluation, particularly to guide further vascular assessment regarding PAD in patients with severe, treatment-requiring aortic stenosis.

Internal validation using bootstrap resampling and stratified five-fold cross-validation supported the performance of the AS-PAD Score within the derivation cohort. Bootstrap validation demonstrated stable model performance after optimism correction, while cross-validation showed acceptable discrimination despite some variability across folds, likely reflecting the limited number of events within each validation subset.

Sensitivity analyses demonstrated that exclusion of BMI ≥ 25 kg/m^2^ or patients who ultimately underwent sAVR had only a limited impact on model performance. Given the potential for incorporation bias related to Fontaine stage, the score was additionally evaluated after removal of this variable. Exclusion of Fontaine stage was associated with a lower AUC; however, discriminatory performance remained moderate and statistically significant. These findings suggest that Fontaine stage contributes to the overall performance of the AS-PAD Score, but that the score is not solely driven by symptomatic PAD severity.

Beyond statistical validation, the clinical relevance of the score is supported by evidence demonstrating that aortic stenosis and peripheral artery disease frequently coexist due to shared mechanisms and risk factors [9]. Moreover, PAD remains under-screened in this population [9]. Taken together, the AS-PAD Score is a practical, statistically sound, and clinically meaningful tool for identifying patients at increased risk of PAD—a population often underdiagnosed according to guidelines [14,16].

### AS-PAD Score in Contemporary TAVI Practice

In patients undergoing TAVI, PAD has been associated with worse outcomes, including increased mortality and a higher risk of access-site complications [3,5,28]. Considering these risks, structured preprocedural PAD screening gains prognostic importance and may support procedural planning, including access route selection.

In TAVI patients, particularly those with relevant PAD, vascular closure can be challenging due to vessel calcification, tortuosity, and small femoral caliber. Contemporary access-site closure strategies include suture-based closure, plug-based closure, and hybrid approaches. Randomized data suggest that a combined suture/plug strategy may reduce vascular complications, shorten time to hemostasis, and decrease bleeding compared with the use of two suture-mediated closure devices alone [29,30].

In cases of suboptimal closure or closure-device failure, additional percutaneous or endovascular management may be required, although the optimal bailout strategy remains dependent on local anatomy, operator experience, and institutional practice [29,30,31].

Evidence comparing specific bailout approaches remains limited, and available data mainly emphasize the importance of technical proficiency and individualized access-site management [32,33]. Comparative studies of primary large-bore access closure have reported broadly similar overall success rates between dual ProGlide and MANTA strategies, although differences have been observed in the need for adjunctive devices and closure failure rates [34,35].

MANTA has also been associated with a slightly higher risk of femoral stenosis in some studies, although this is rarely clinically significant [36]. Randomized data comparing dual ProGlide with hybrid ProGlide plus Angio-Seal strategies suggest that hybrid closure may reduce vascular complications and device failure rates, while registry data support MANTA as a feasible rescue option after suture-based closure failure [30,33]. Nevertheless, the present study was not designed to compare closure systems or to determine the optimal access-site closure strategy. Although device cost and the need for adjunctive closure devices may influence procedural decision-making, these considerations are beyond the primary scope of this analysis [34,37,38]. The AS-PAD Score should therefore be interpreted as a complementary clinical tool for estimating significant PAD and contextualizing potential femoral access-site risk, rather than as a substitute for CT-based access assessment or as an independent predictor of major vascular complications.

Our findings are consistent with the 2024 ESC Guidelines, which emphasize the need for systematic risk assessment for PAD in cardiovascular patients [14]. Similar diagnostic aspects were already addressed in the 2017 ESC/ESVS Guidelines, underlining the ongoing relevance of vascular risk stratification in cardiovascular care [15]. Importantly, the AS-PAD Score is not intended to replace guideline-recommended contrast-enhanced CT assessment of the aorta and iliofemoral access route, which remains essential for determining procedural feasibility and access strategy. Rather, the score should be interpreted as a complementary clinical risk-stratification tool that may help contextualize the probability of significant PAD and potential access-site risk alongside standard imaging findings. This study was not designed to demonstrate superiority over CT-based vascular assessment, and the incremental value of the AS-PAD Score beyond CT-derived anatomical parameters should be evaluated in future prospective studies. In selected patients in whom CT suggests that transfemoral TAVI is technically feasible, but residual uncertainty regarding procedural risk remains, the score may support Heart Team discussion and contribute to individualized preprocedural planning.

## 5. Limitations

Despite encouraging model performance, several limitations should be acknowledged. First, this was a single-centre retrospective derivation study with a limited sample size and a limited number of significant PAD events. Accordingly, the present findings should be interpreted as derivation and internal validation results and do not yet establish external generalizability. Future studies should externally validate the AS-PAD Score in larger, independent multicenter TAVI cohorts and evaluate its potential integration into clinical decision-making workflows. Furthermore, the limited number of patients with significant PAD in relation to the five score components represents an important methodological limitation. This low event-to-variable ratio may increase the risk of model overfitting, coefficient instability, and optimistic estimates of discriminatory performance. In addition, the use of backward stepwise regression may have influenced the final selection and weighting of predictors included in the AS-PAD Score and may affect the stability of the model. As with other data-driven variable selection approaches, this can limit reproducibility and may lead to overly optimistic estimates of model performance. Although apparent calibration and decision-curve analysis were performed, external calibration and prospective clinical utility assessment remain lacking and should be addressed in future studies. Taken together, these limitations suggest that the AS-PAD Score should be regarded as a hypothesis-generating tool derived from a single-center cohort. Moreover, the incremental value of the AS-PAD Score beyond CT-derived anatomical parameters was not assessed and should be evaluated in future prospective cohorts. Because only minor femoral access-site closure-device failures occurred in our cohort, the association between the AS-PAD Score and post-TAVI access-site outcomes could only be assessed for minor closure-related events. No major PAD-related vascular complications were observed. Moreover, this exploratory analysis was vulnerable to residual confounding, as closure-device failure may be influenced by several local anatomical and procedural factors that were not fully adjusted for in the present study, including femoral artery diameter, calcification distribution, puncture height, sheath-to-femoral artery ratio, operator technique, ultrasound-guided access, valve platform, and closure strategy. Therefore, the observed association should not be interpreted as evidence that the AS-PAD Score independently predicts major vascular complications after TAVI. This should be further evaluated in larger prospective cohorts with systematic assessment of CT-derived access parameters and procedural variables.

## 6. Conclusions

The AS-PAD Score may serve as an adjunctive clinical tool for estimating the likelihood of significant PAD in patients with severe AS undergoing TAVI evaluation. Its association with femoral access-site closure-device failure should be interpreted as exploratory and hypothesis-generating. Further external validation is required to confirm its role alongside standard CT-based vascular assessment.

## Figures and Tables

**Table 1:** Baseline characteristics of the study population.

Variable	*n* = 138	%
Age, years	81.2 ± 8.01	
Male	62	44.93%
Female	76	55.07%
CAD	98	71.01%
One-vessel CAD	35	25.36%
Two-vessel CAD	25	18.12%
Three-vessel CAD	38	27.54%
Non-significant CAD	13	9.42%
Significant CAD	85	61.59%
PAD	88	63.77%
Iliac	58	42.03%
Femoropopliteal	73	52.90%
Infrapopliteal	85	61.59%
Fontaine stage I	64	46.38%
Fontaine stage IIa	7	5.07%
Fontaine stage IIb	11	7.97%
Fontaine stage III	4	2.90%
Fontaine stage IV	2	1.45%
Non-significant PAD	53	38.41%
Iliac	46	33.33%
Femoropopliteal	50	36.23%
Infrapopliteal	53	38.41%
Significant PAD	35	25.36%
Iliac	12	8.70%
Femoropopliteal	23	16.67%
Infrapopliteal	32	23.19%
CAS	56	40.58%
Unilateral	42	30.43%
Bilateral	14	10.14%
Significant CAS	20	14.49%
Unilateral	16	11.59%
Bilateral	4	2.90%
Cardiovascular risk factors		
Arterial hypertension	124	89.86%
Diabetes mellitus	51	36.96%
Chronic kidney disease	32	23.19%
Current or former smoking	79	57.25%
<10 pack years	18	13.04%
10–20 pack years	31	22.46%
>20 pack years	30	21.74%
BMI	27.1 ± 4.83	
BMI <18.5 kg/m^2^	1	0.72%
BMI 18.5–24.9 kg/m^2^	49	35.51%
BMI ≥ 25 kg/m^2^	82	59.42%
BMI 25–29.9 kg/m^2^	51	36.96%
BMI 30–34.9 kg/m^2^	20	14.49%
BMI 35–39.9 kg/m^2^	10	7.25%
BMI ≥40 kg/m^2^	1	0.72%
BMI: not stated	6	4.35%
Lp(a) *, nmol/L	47.8 (18.3–171.7)	
IL-6 *, pg/mL	10.26 (6.00–16.25)	
hs-CRP *, mg/L	3.87 (1.19–7.61)	
Total Cholesterol, mg/dL	168.00 (138.00–203.00)	
Triglycerides, mg/dL	112.50 (85.50–160.50)	
LDL, mg/dL	93.45 (63.93–127.38)	
HDL, mg/dL	51.90 (39.58–61.60)	

CAD = coronary artery disease; PAD = peripheral artery disease; CAS = carotid artery stenosis; BMI = body mass index; Lp(a) = lipoprotein(a); IL-6 = interleukin-6; hs-CRP = high-sensitivity C-reactive protein; LDL = low-density lipoprotein; HDL = high-density lipoprotein. Data are presented as mean ± standard deviation, median (Q1–Q3), or n (%), as appropriate. Percentages refer to the total study population (*n* = 138). Significant CAD was defined as ≥50% stenosis in at least one coronary artery, significant PAD as ≥50% stenosis in at least one lower-extremity artery, and significant CAS as ≥50% stenosis in at least one carotid artery. PAD distribution is reported according to iliac, femoropopliteal, and infrapopliteal involvement; these vascular territories are not mutually exclusive. * Missing values were present for BMI (*n* = 6), Lp(a) (*n* = 5), hs-CRP (n = 10), and IL-6 (*n* = 16). Lp(a) measurements were available for all patients with significant PAD.

**Table 2:** Univariable and multivariable logistic regression analysis for final AS-PAD Score.

Variable	Univariable OR (95% CI)	*p*-Value	Multivariable OR (95% CI) *	*p*-Value
Age > 75 years	2.51 (1.42–7.39)	0.031	2.43 (1.05–5.70)	0.039
Diabetes mellitus	3.01 (1.38–6.57)	0.005	3.18 (1.29–7.83)	0.012
BMI ≥ 25 kg/m^2^	0.48 (0.21–1.08)	0.070	0.34 (0.14–0.82)	0.016
Lp(a) >50 mg/dL (105 nmol/L)	1.68 (1.06–2.67)	0.028	1.42 (1.01–2.36)	0.044
Fontaine stage (I–IV) **	—	—	—	—

* Multivariable results derived from the final model using backward Wald elimination. ** Fontaine stage was not modelled as a binary or continuous predictor but incorporated directly into the scoring system to reflect clinical severity.

**Table 3:** Bootstrap-based internal validation and apparent calibration of the AS-PAD Score.

Parameter	Value
Apparent AUC	0.804
Optimism-corrected AUC	0.805
Brier score	0.141
Optimism-corrected Brier score	0.146
Apparent calibration intercept	0.000
Apparent calibration slope	1.000
Bootstrap-corrected calibration intercept	0.009
Bootstrap-corrected calibration slope	1.003

AUC = area under the curve. Internal validation was performed using 1000 bootstrap iterations with optimism correction. Calibration was additionally assessed using the Brier score and a 95% calibration belt.

**Table 4:** Five-fold cross-validation performance of the AS-PAD Score.

Fold	Validation Sample, n	Significant PAD Events, n	Non-Events, n	AUC
Fold 1	28	7	21	0.939
Fold 2	28	7	21	0.905
Fold 3	28	7	21	0.636
Fold 4	27	7	20	0.829
Fold 5	27	7	20	0.661
Mean ± SD	—	—	—	0.794 ± 0.139
Median (IQR)	—	—	—	0.829 (0.661–0.905)
Pooled cross-validated AUC	138	35	103	0.789 (95% bootstrap CI: 0.696–0.875)

AUC = area under the curve; CI = confidence interval; IQR = interquartile range; PAD = peripheral artery disease; SD = standard deviation. Five-fold cross-validation was performed using out-of-fold predicted probabilities from logistic regression models with the AS-PAD Score as a continuous predictor. The pooled cross-validated AUC was calculated from all out-of-fold predictions, and its 95% CI was estimated using bootstrap resampling.

**Table 5:** Sensitivity analyses of the AS-PAD Score.

Analysis	AUC (95% CI)	*p*-Value
Original model	0.804 (0.722–0.887)	<0.001
Without Fontaine stage	0.701 (0.600–0.801)	<0.001
Without BMI ≥25 kg/m^2^	0.789 (0.704–0.875)	<0.001
Without sAVR patients	0.785 (0.695–0.875)	<0.001

AUC = area under the curve; CI = confidence interval; sAVR = surgical aortic valve replacement. *p*-values refer to the test of the AUC against 0.5.

## Data Availability

The data presented in this study are available on reasonable request from the corresponding author. The data are not publicly available due to privacy and ethical restrictions.

## Abbreviations

The following abbreviations are used in this manuscript:

ACC	American College of Cardiology
AHA	American Heart Association
AS	Aortic stenosis
AS-PAD	Aortic Stenosis–Peripheral Artery Disease Score
AUC	Area under the curve
AVA	Aortic valve area
BMI	Body mass index
CI	Confidence interval
CTA	Computed tomography angiography
EACTS	European Association for Cardio-Thoracic Surgery
EAS	European Atherosclerosis Society
ESC	European Society of Cardiology
ESVS	European Society for Vascular Surgery
hs-CRP	High-sensitivity C-reactive protein
IL-6	Interleukin-6
Lp(a)	Lipoprotein(a)
MANTA	Collagen-based vascular closure device
MRA	Magnetic resonance angiography
NHANES	National Health and Nutrition Examination Survey
OR	Odds ratio
PAD	Peripheral artery disease
ROC	Receiver operating characteristic
sAVR	Surgical aortic valve replacement
SPSS	Statistical Package for the Social Sciences
TAVI	Transcatheter aortic valve implantation
VARC-3	Valve Academic Research Consortium-3
